# Characterization of a new B-ALL cell line with constitutional defect of the Notch signaling pathway

**DOI:** 10.18632/oncotarget.24836

**Published:** 2018-04-06

**Authors:** Paul Takam Kamga, Giada Dal Collo, Giulio Bassi, Martina Midolo, Massimo Delledonne, Marco Chilosi, Massimiliano Bonifacio, Mauro Krampera

**Affiliations:** ^1^ Stem Cell Research Laboratory, Section of Hematology, Department of Medicine, University of Verona, Verona, Italy; ^2^ Department of Biotechnology, University of Verona, Verona, Italy; ^3^ Personal Genomics S.R.L., Verona, Italy; ^4^ Section of Pathology, Department of Diagnostics and Public Health, University of Verona, Verona, Italy

**Keywords:** Notch signaling, B-acute lymphoblastic leukemia, B-ALL, Alagille syndrome, ALGS

## Abstract

Notch signaling contribution to B-cell acute lymphoblastic leukemia (B-ALL) development is still under investigation. The serendipitous onset of B-ALL in a patient affected by the germinal Notch mutation-dependent Alagille syndrome allowed us to establish a B-ALL cell line (VR-ALL) bearing a genetic loss of function in components of Notch signaling. VR-ALL is a common-type B-ALL cell line, grows in conventional culture medium supplemented with 10% serum, and gives rise, once injected into immunodeficient NOG mice, to a mouse xenograft model of B-ALL. Exome sequencing revealed deleterious mutations in some components of Notch signaling, including Jagged1, Notch1, and Notch2. In addition, VR-ALL is sensitive both *in vitro* and *in vivo* to γ-secretase inhibitors (GSIs) as well as conventional anti-leukemic drugs. For all these reasons, VR-ALL may help to gain more insights into the role of Notch signaling in B-ALL.

## INTRODUCTION

Acute lymphoblastic leukemia (ALL) is the most common cancer in children, adolescents and young adults. Many aberrations in some signaling pathways are involved in ALL pathogenesis; amongst them, gain-of-function mutations in *NOTCH1* gene have been described in more than 50% of T-cell acute lymphoblastic leukemia (T-ALL) cases, thus unraveling the role of Notch-mediated oncogenesis in lymphoid tissues. Enhanced Notch1 activity in hematopoietic stem/progenitor cells leads to T-ALL-like disease in mice, while genetic loss of function or the use of pharmacological Notch signaling inhibitors, such as γ-secretase inhibitors (GSIs), sensitize T-ALL cells to glucocorticoid treatment. Notch signaling is an evolutionary conserved pathway, consisting of 4 receptors (Notch1-4) and 5 ligands (Jagged1, Jagged2, DLL-1, DLL-3 and DLL-4). Ligand binding induces γ-secretase-mediated cleavage of Notch intracellular domain (NICD), which is transferred into the nucleus and interacts with the DNA-binding protein RBP-J, thus inducing the expression of downstream target genes, i.e. Hes1 and Deltex1 [1]. Notch signaling dysregulation is involved in many malignancies, including ALL [2, 3]. Considering the number and complexity of the interactions amongst Notch and several other intracellular signaling pathways involved in cell survival, proliferation and apoptosis, the precise role of Notch pathway can be hardly identified during the neoplastic lymphoid cell development. Particularly, the role of Notch signaling in B-cell acute lymphoblastic leukemia (B-ALL) pathogenesis is still under investigation due to the lack of specific mutations. A relatively large number of B-ALL cell lines have been established to investigate the contribution of signaling proteins to the disease. In this study, we describe a new cell line (VR-ALL) derived from the bone marrow sample of a patient affected by both B-ALL and Alagille syndrome (ALGS), and carrying multiple aberrations in Notch components.

ALGS (OMIM 118450), also known as Alagille–Watson syndrome or arteriohepatic dysplasia, is an autosomal dominant genetic disease affecting Notch signaling pathway and involving different organs, such as liver (lack of intra hepatic bile ducts leading to chronic cholestasis), heart (malformations affecting the pulmonary outflow tract and vasculature), skeleton (butterfly thoracic vertebrae due to fusion failure of the anterior vertebral arches; typical facies with a broad forehead; digital fusiform shape with hypoplasia of terminal phalanges), eyes (pigmentary retinopathy, cataracts, posterior embryotoxon and/or anterior segment abnormalities), kidneys (renal dysplasia), and central nervous system (intracranial bleeding) [4, 5]. Estimated prevalence, on the basis of the presence of neonatal hepatic abnormalities, is 1:70,000; however, the presence of variable expression, reduced penetrance, new mutations (~60%) and the possibility of germline mosaicism likely determines the underestimation of the disease frequency. Most cases (~97%) are caused by haploinsufficiency of Notch signaling pathway, mostly due to mutations or (less often) locus deletions of the *JAGGED1* gene (20p11.2-20p12). Very rarely (<1%) *NOTCH2* mutations are responsible for the disease, with prevalent renal involvement [4, 5].

Here we performed a cellular and molecular characterization of VR-ALL cell line, revealing that VR-ALL is a B-ALL cell line growing both *in vitro* and *in vivo* in NOG mice. VR-ALL cell line is sensitive to Notch modulators and conventional chemotherapeutic agents, such as cytarabine, doxorubicin and dexamethasone. The availability of this new cell line with a natural loss of function in Notch pathway will be helpful to assess the contribution of Notch signaling in the pathogenesis of B-ALL and its chemosensitivity.

## RESULTS

### B-ALL cell processing and cell line stabilization

**Figure 1:**
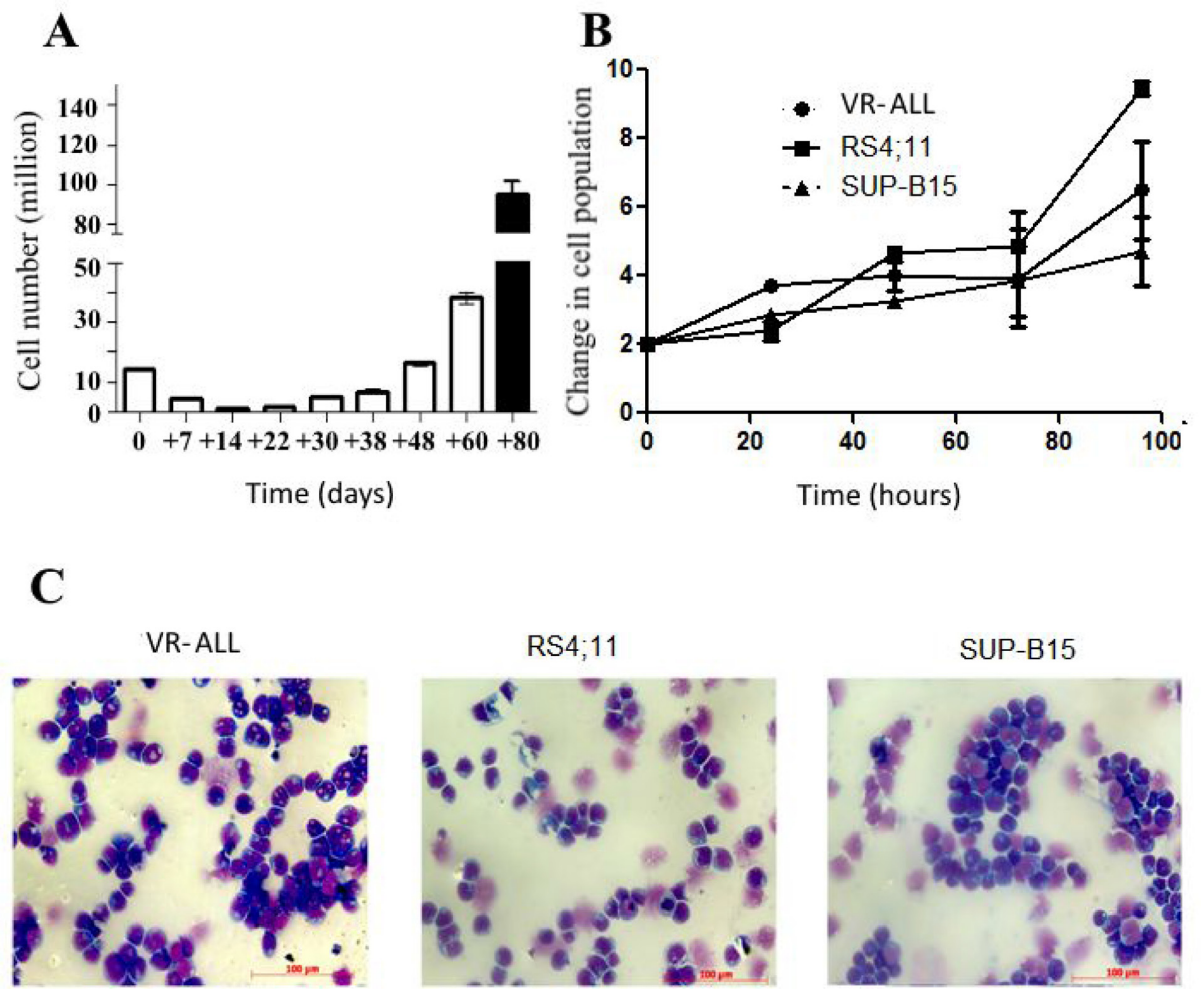
Growth and morphological patterns of VR-ALL (**A**) Initial proliferation rate of VR-ALL cells isolated from the ALGS patient. Blast cells derived from the patient were grown in RPMI with 10% FBS, cell count was performed routinely. (**B**) Proliferation rate of VR-ALL cells 3 years following isolation; cells were grown in RPMI with 10% FBS, cell count was performed every 24 hours. Data are reported as mean ± SEM of 4 independent experiments performed in duplicate. (**C**) Cell morphology of B-ALL cell lines stained with May Grunwald-Giemsa staining and observed using Axiovert Z1 Observer Microscope (Zeiss).

Mononuclear cells from bone marrow samples of the ALGS/B-ALL patient at diagnosis were separated with density gradient centrifugation and cultured in complete RPMI 1640 at 37° C, 5% CO_2_. Cell number was relatively stable till day 38 (Figure 1A). Then cells started to grow exponentially and were successfully expanded and sub-cultured (Figures 1A, 1B). Cell growth capability was maintained after short (−80° C) or long-term (liquid nitrogen) freezing and for more than 1 year of culture; consequently, this homogeneous cell population was considered as a cell line (VR-ALL).

### VR-ALL cell line characterization

Cells were negative for Epstein–Barr virus and mycoplasma (data not shown), displayed a normal male karyotype (46, XY) and were negative for BCR-ABL fusion transcript. VR-ALL cell line features were compared with those of two other well-known B-ALL cell lines, i.e. RS4;11 and SUP-B15 [6, 7] through flow cytometric analysis (Table 1) and May-Grünwald Giemsa staining (Figure 1C). In line with the immunophenotypic profile of the initial leukemic cells from the ALGS patient, VR-ALL cell line displayed multiple B-cell lineage markers, such as CD10, CD20, CD22, CD34, CD38, CD45, CD58, cyCD79a and TdT, and lack of myeloid markers, such as CD13 and myeloperoxidase (MPO), as well as T cell markers (Table 1). VR-ALL and the two other B-ALL cell lines shared a similar morphologic pattern, i.e. blast cells of small to medium size with high nuclear-cytoplasmic ratio, one or more nucleoli and many intracytoplasmic vacuoles (Figure 1C).

**Table 1:** Characterization of blast cells derived from bone marrow aspirate of the ALGs/B-ALL patient

SSC/CD19+	ALGS patient	VR-ALL	RS4;11	SUP-B15
**CD10**	++	++	-	+++
**CD13**	-	+/−	-	-
**CD15**	+/−	+	+++	+
**CD20**	+	+	-	+
**CD22**	++	++	+++	+++
**CD33**	+	+	+	-
**CD34**	++	+	-	+++
**CD38**	++	+	++	++
**CD45**	+	+	++	-
**CD58**	++	+	++	+
**CD66c**	++	-	-	-
**CD133**	-	**na**	**na**	**na**
**cyCD79a**	++	+	-	+
**cyIgμ**	-	+	-	+
**cytCD3**	-	**na**	**na**	**na**
**MPO**	-	-	-	-
**NG2**	-	+	-	-
**TdT**	+	+	+	+

Flow cytometry analysis of patient's blast cells using fluorescent conjugated antibodies specific to extracellular or/and intracellular protein markers. For each antibody, the mean of fluorescence was normalized to the mean of fluorescence obtained with its specific isotype conjugated to the same fluorescent marker. “na”: not available.

### VR-ALL cell line proliferation and engraftment potential

**Figure 2:**
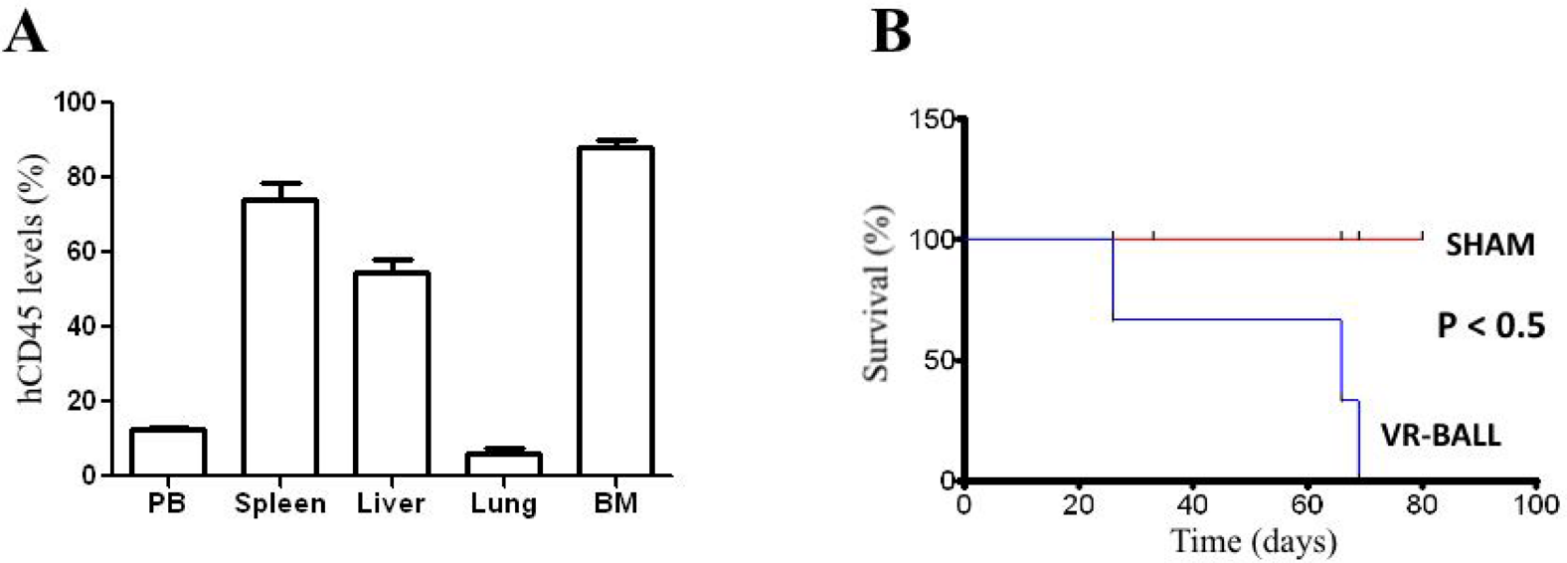
Engraftment of VR-ALL cells into NOG mice 5 × 10^6^ VR-ALL cells were injected via the tail vein into 8 to 12 week-old mice. Mice were either (**A**) sacrificed (*n* = 4) at 8 weeks following the initial injection of VR-ALL cells to evaluate leukemic burden in organs as levels of hCD45+ cells, or (**B**) monitored (*n* = 4) for their survival. “Sham”: control or naive mice.

VR-ALL cell line grew easily in RPMI or IMDM supplemented with 10% FBS, 1% L-Glutamine and 1% Penicillin/Streptomycin, similarly to RS4;11 and SUP-B15 cell lines. VR-ALL cells were seeded at a density of 0.5-1 × 10^6^/ml. Population doubling time of VR-ALL cells was about 56 hours, displaying similar proliferation rate with SUP-B15 cells, but slower than RS4;11 (Figure 1B). Tumorigenicity was assessed *in vivo* through engraftment assay into immune-deficient mice. For this purpose, 5 × 10^6^ cells were injected into NOG mice. Eight weeks following cell injection, mice were sacrificed and the organs were analyzed through flow cytometry for the presence of human leukemic cells (hCD45+ cells). Leukemic cell distribution in animal organs included peripheral blood (PB) (10–15%), spleen (60–70%), liver (40–60%), lung (5–10%) and bone marrow (70–90%) (Figure 2A). Mice receiving VR-ALL cell line (*n* = 4) died within 70 days (Figure 2B).

### Genomic pattern of VR-ALL cell line

To identify the putative causative variants of ALGS and B-ALL in the initial patient affected by both diseases, we sequenced the whole exome of the patient and the cell line. Among the most notable putative pathogenetic events, a p.P871R substitution in *JAGGED1* was observed. *JAGGED1* is located in the 20p12 locus and is mutated in 94% of the individuals affected by ALGS [4, 5]. We also found a missense variant in *NOTCH2*, the second gene whose mutations are associated with type 2 ALGS [4]. To exclude mutations associated only with ALGS, we performed a whole genome sequencing of 4 other patients suffering from B-ALL only. None of these patients presented any mutation associated with ALGS, such as aberrations in *JAGGED1* or/and *NOTCH2* (data not shown). In addition, we also identified mutations in genes already reported for their association with T-ALL and/or B-ALL [8]; notably, missense mutations were observed in *PAX5*, *NOTCH1*, *NOTCH3*, *EPHA2, NCOR1* and *PIK3C2B* (Table 2). To support the results obtained and identify further putative pathogenetic variants for B-ALL, we turned to disease-network analysis. We used two algorithms: Endeavour [9] and ToppGene [10]. The results of gene prioritization are shown in Table 2. Interestingly, we observed a similar pattern of mutation events between initial patient cells and the VR-ALL line (Table 2), thus confirming that the cell line effectively originated from the initial patient.

**Table 2:** List of prioritized genes

Rank of genes	ALGS-PATIENT	VR-ALL cells
* 1*	*NOTCH1*	*NOTCH1*
* 2*	*NOTCH3*	*NOTCH3*
* 3*	*JAGGED1*	*EPHA2*
* 4*	*EPHA2*	*PAX5*
* 5*	*PAX5*	*CHUK/ERLIN1*
* 6*	*PTPN11*	*STAT1*
* 7*	*CHUK/ERLIN1*	*HD*
* 8*	*STAT1*	*MST1*
* 9*	*HD*	*LAMA3*
*10*	*MST*	*KRT18*
*11*	*LAMA3*	*TF*
*12*	*KRT18*	*NUMA1*
*13*	*TF*	*LTBP1*
*14*	*NUMA*	*NCOR1*
*15*	*LTBP1*	*SERPINA5*
*16*	*NCOR1*	*BCLAF1*
*17*	*BCLFAP*	*PABPC1*
*18*	*SERPINA5*	*PIK3C2B*
*19*	*PABPC1*	*LIG1*
*20*	*LIG1*	*PARP1*
*21*	*PIK3C2*	*JAGGED1*

The rank of genes with potentially damaging mutations in different disease prediction algorithms and the combined results.

### Notch signaling status in VR-ALL cells

**Figure 3:**
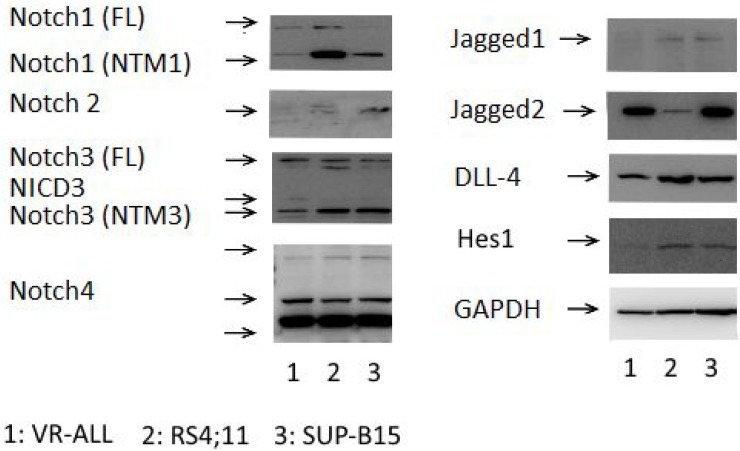
Notch expression and activation in B-ALL samples Immunoblot of VR-ALL cells and B-ALL cell lines RS4;11 and SUP-B15, probed for Notch1-4, Jagged1-2, DLL4, Hes1 and GADPH. Data are representative of 6 independent experiments. “FL”: Full Length, “NTM”: Notch Transmembrane Domain, “NICD”: Notch Intracellular Domain.

**Figure 4:**
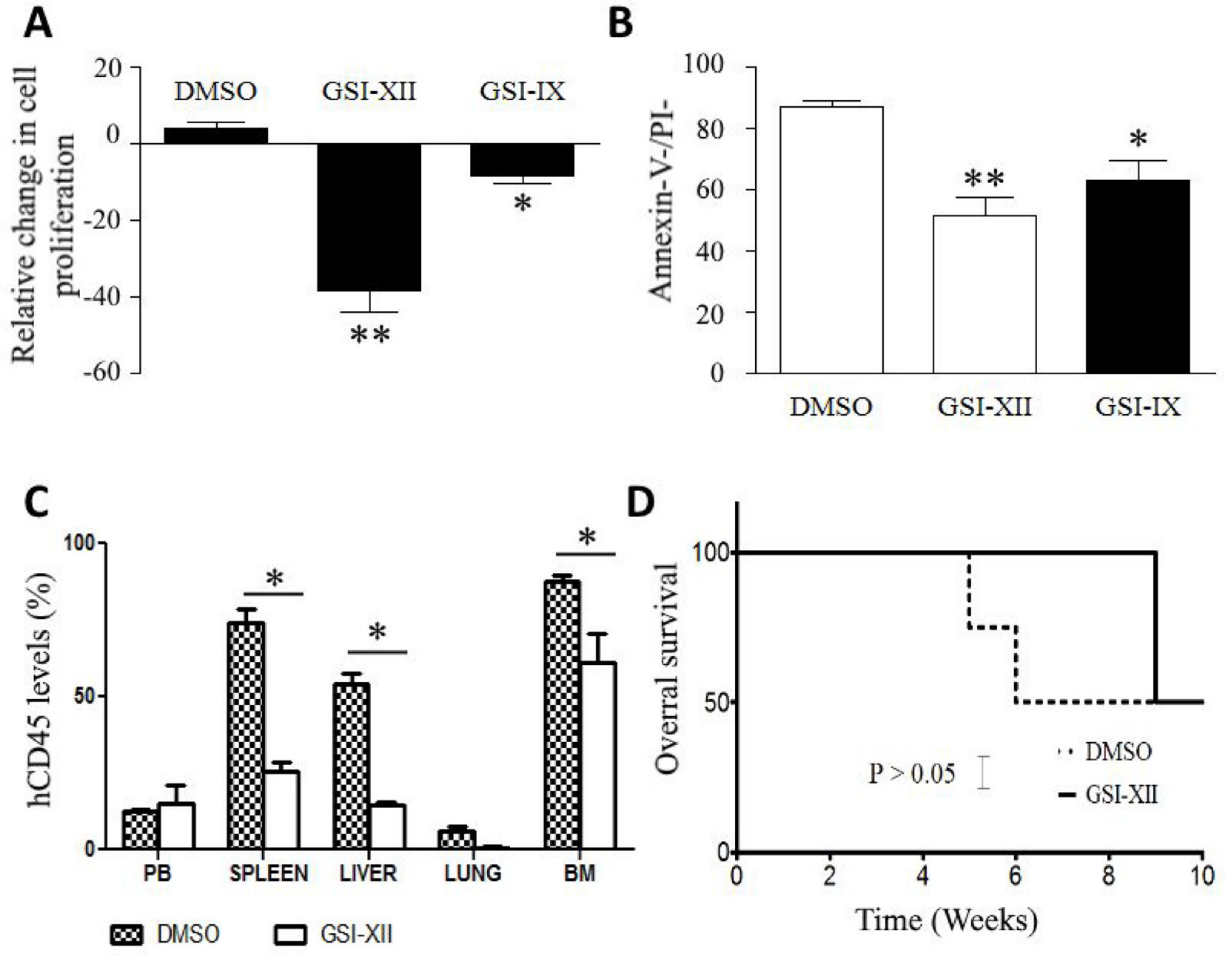
GSIs reduces VR-ALL cell viability (**A**) Relative proliferation of VR-ALL cells stained with CFSE and treated for 2 days with GSI-XII (10 μM) and GSI-IX (15 μM); CFSE dilution was analyzed though flow cytometry and expressed as relative proliferation. Data are reported as mean ± SEM of 4 independent experiments performed in duplicate ^**^p < 0.01, ^**^p < 0.001. (**B**) Apoptosis levels in VR-ALL cells treated for 2 days with GSI-XII (10 μM) and GSI-IX (15 μM); cells were stained with Annexin-V and propidium before analysis through flow cytometry. Data are reported as mean ± SEM of 4 independent experiments performed in duplicate ^**^p < 0.01, ^***^p < 0.001. (**C–D**) Effect of GSI-XII administration on leukemic burden and mouse overall survival after injection of VR-ALL cell line (DMSO: solvent of GSI-XII, negative control).

Western blot analysis showed higher levels of Notch-1, Notch-3, Notch-4, Jagged-2 and DLL-4 expression in VR-ALL cell line (Figure 3). In general, molecular aberrations leading to ALGS, i.e mutations in *JAGGED1* and *NOTCH2,* are mostly associated with decreased Notch signaling [11]. Consistently, Western blot analysis of VR-ALL cells revealed low levels of Jagged-1 and absence of Notch-2 proteins (Figure 3). Importantly the Notch target gene HES1 was slightly detectable in VR-ALL compared to RS4;11 and SUP-B15, suggesting that Notch signal could be effectively inactivated in VR-ALL cells (Figure 3). Surprisingly, the treatment of VR-ALL cells with Notch signaling inhibitors, i.e. GSI-IX and GSI-XII, reduced VR-ALL cell proliferation and induced cell death (Figure 4A, 4B). In the *in vivo* mouse xenograft model of VR-ALL, the treatment of mice with GSI-XII reduced leukemic burden in various organs (Figure 4C), but did not improve overall survival (Figure 4D). As the pathway is not activated, as shown by the low levels of Hes1 expression (Figure 3A), sensitivity of VR-ALL to GSIs could reflect either a Notch-independent activity of these drugs [12] or a non-CSL coupling of Notch signaling in VR-ALL cells [13].

### VR-ALL cell line is sensitive to anti-leukemic agents

As cell lines are a powerful tool to evaluate the activity of drug candidates [14], we assessed *in vitro* the sensitivity of VR-ALL cell line to some classic anti-leukemic agents, such as Cytarabine, Dexamethasone, Doxorubicin, Bortezomib and MG132. Increasing concentrations of these pharmacological agents determined significant and dose-dependent decrease in VR-ALL cell viability. The IC50 values for each drug are shown in Table 3. Noteworthy, we observed that VR-ALL cells were less sensitive to the treatment with Dexamethasone than the two other B-ALL cell lines. Accordingly, the pretreatment of the Alagille patient with steroids failed to induce a significant decrease of the leukemic burden. Glucocorticoid resistance has been described as a hallmark of treatment failure in B-ALL [14]. Thus, VR-ALL could be a good tool to investigate the mechanisms of B-ALL relapse determined by glucocorticoid refractoriness.

**Table 3:** Sensitivity of B-ALL cell lines to drugs

Drugs	VR-ALL (a)	RS4 ;11 (b)	SUP-B15 (c)		*P*-values
Bortezomid				a vs b	*P* = 0.01
	3.11 × 10^−9^	1.5 × 10^−9^	4.1 × 10^−9^	a vs c	*P* = 0.5
Cytarabine				a vs b	*P* = 0.11
	5.3 × 10^−7^	8.4 × 10^−7^	6.4 × 10^−7^	a vs c	*P* = 0.15
Dexamethasone				a vs b	*P* = 0.03
	1.2 × 10^−8^	3.1 × 10-9	4.2 × 10^−9^	a vs c	*P* = 0.04
Doxorubicin				a vs b	*P* = 0.11
	1.6 × 10^−8^	1.9 × 10-8	2.1 × 10^−8^	a vs c	*P* = 0.03
MG132				a vs b	*P* = 0.01
	1.8 × 10^−7^	1.2 × 10^−7^	1.7 × 10^−7^	a vs c	*P* = 0.17

Cells were cultured for 48 h with increasing concentration of each drug. Then cell viability was assessed through MTS assay. The effective concentration to induce 50% reduction of B-ALL cells viability (IC50) derived from the equations that best fit the linear range of the dose-response curve. Each experimental condition was done in 8 replicates and repeated at least two times. “vs”: versus.

## DISCUSSION

The availability of disease-specific cell lines, as versatile and informative *in vitro* models, offers the unique opportunity to analyze the pathobiology of human malignancies [15]. In this study, we described the biological features of a new human B-ALL cell line derived from bone marrow mononuclear cells of an ALGS patient affected by BCR-ABL-negative, common B-ALL. Flow-cytometric characterization revealed the B-ALL immunophenotype of VR-ALL cell line, with some antigen features in common with the pre-B-ALL cell line RS4;11. VR-ALL cell line retained after more than three years from initial diagnosis, the same antigen pattern observed in the primary sample from the patient, thus proving cell stability in culture.

The autonomous and growth factor-independent proliferation of the cultured cells is an important property of the cell lines used in cancer research [16]. VR-ALL cells were grown in normal RPMI medium supplemented with 10% FBS, displaying a proliferation rate almost equivalent to RS4;11 and SUP-B15 cell lines. In addition, we successfully obtained B-ALL xenograft models by injecting VR-ALL cells in the tail vein of NOG mice, thus highlighting the tumorigenic anchorage capacity of the cell line into immunodeficient mice [17]. The above-mentioned features are both fundamental for the evaluation of drug sensitivity *in vitro* and *in vivo*. In fact, *in vitro* treatment of cell lines with active compounds represents the early phase of drug development [18] and provides additional prognostic information in ALL [19], while the cell line-based xenograft models, due to their high reproducibility, reduce the number of potential bias interfering with the evaluation of drug sensitivity *in vivo* [20].

VR-ALL cell line derived from a patient who was refractory to steroid treatment and eventually relapsed. When compared to the B-ALL cell lines RS4;11 and SUP-B15, VR-ALL cell line did not display significant difference in drug sensitivity, except for Dexamethasone. Resistance to glucocorticoids has been reported as a relapse hallmark in ALL, and resistance to glucocorticoids *in vitro* is associated with unfavorable prognosis [14]. Growing evidence supports the concept that Notch inhibition through GSIs can abrogate glucocorticoid resistance. Mechanistically, GSIs increase transcriptional upregulation of the glucocorticoid receptor and target genes, thus promoting glucocorticoid-mediated apoptosis in T-ALL primary cells and T-ALL cell lines, such as CUTTL1, KOPT1 and T-ALL1 [21, 22]. To our knowledge, VR-ALL is the first B-ALL cell line carrying a background of ALGS, an inherited disease characterized by loss-of-function in Notch components. Some rare and inherited diseases are characterized by molecular lesions promoting cancer diseases [23]. For example, RASopathies, such as Noonan syndrome, Neurofibromatosis 1 and Leopard syndrome, are a subtype of developmental diseases characterized by mutations in genes encoding for components of the Ras/MAPK pathway (*NF1*, *PTPN1*, *SOS1, RAF1, KRAS, NRAS, SHOC2, CBL*) [24, 25]. RASopathies are associated to higher risk to develop AML, ALL and Myeloproliferative/Myelodysplastic syndromes [26–28]. In VR-ALL cells, the analysis of Notch expression pattern showed that the pathway was present but poorly active, as demonstrated by the absence of Notch target gene *HES1*, thus revealing that the mutations observed in the components of Notch signaling had led to a loss-of-function effect, switching off the pathway signaling. This appears in contrast to the higher sensitivity of VR-ALL cells to GSI-XII, suggesting a Notch independent action of GSIs [12]. Nevertheless, gamma-secretase complexes have many other substrates besides Notch involved in cell viability, including CD44, β-catenin, GSK-3β, and N-cadherin [29–31]. Their inhibition could then be achieved by using GSIs, thus leading to growth arrest in a Notch-independent manner. On the other hand, evidence from studies revealed that GSIs can display anti-leukemic activity by a direct inhibition of the proteasome [32, 33]. Consistently, Meng *et al.* proposed that some GSIs may reduce B-ALL cell viability through proteasome inhibition [32, 33]. Accordingly, we observed that VR-ALL cells were highly sensitive to some proteasome inhibitors, such as MG132 and Bortezomib. Hence, VR-ALL cells represent a tool that may help to gain more insights into the Notch-independent anti-leukemic activity of GSIs. In addition, we and other groups have previously demonstrated that the expression and activation of the Notch pathway could also depend on the interaction with stromal cells [34, 35]. Therefore, 2D-co-culture of VR-ALL cells with stromal cells may give additional information on Notch signaling expression, activation and contribution to leukemic cell support; more generally, VR-ALL cell line may help to understand B-ALL pathogenesis and represents a good tool to better unravel the mechanistic role of Notch signaling in B-ALL.

## MATERIALS AND METHODS

### Case report, samples and cell lines

Peripheral blood and bone marrow samples were collected from a 20-year-old man with ALGS, mainly with liver, heart and skeleton involvement, who developed a Philadelphia-negative, normal karyotype (46, XY), common B-ALL (CD19+++, CD10++, CD13–, CD15+/−, CD20+, CD22++, CD33+, CD34++, CD38++, CD45+, CD58++, CD66c++, CD133–, cyμ–, cytCD3–, MPO–, NG2- and TdT (Table 1). Because of parents (who had the parental authority) opposition, the patient did not start the intensive chemotherapy, but received only pretreatment with steroids (with partial response) and some doses of Vincristine, achieving a short-term hematologic complete remission before going to overt disease progression and exitus. All samples were collected before treatment, as approved by the Ethical Committee of *Azienda Ospedaliera Universitaria Integrata Verona Italia* (N. Prog. 1828, May 12, 2010 - *‘Institution of cell and tissue collection for biomedical research in Onco-Hematology’*). Leukemic cells from patient peripheral blood displayed autonomous and external growth factor-independent proliferation in culture and were considered as a cell line, referred to as the VR-ALL cell line. Human B-ALL cell lines including VR-ALL, RS4;11, SUP-B15 were cultured in complete RPMI 1640 (RPMI supplemented with 10% FBS, 1% L-Glutamine and 1% Penicillin/Streptomycin). All cell lines were Mycoplasm-free. Cell morphology was assessed with May Grunwald-Giemsa staining using Axiovert Z1 Observer Microscope (Zeiss).

### Western blotting

Cells were lysed with an appropriate amount of RIPA buffer (25 nM Tris pH 7.6, 150 mM NaCl, 1% NP40, 1% Na-deoxycholate, 0.1% SDS) supplemented with complete Protease Inhibitor (Roche) and 1 mM Na3VO4. Proteins were quantified using BCA protein assay kit (Thermo Scientific) and separated on 10% or 12% polyacrylamide gel. Subsequently, proteins were transferred onto nitrocellulose membrane (GE Healthcare), labeled with the appropriate antibody and acquired by LAS4000 (GE Healthcare) instrument. GAPDH was used as loading control.

### MTT viability assay

To study the specific relative basal sensitivity of B-ALL cell lines to Notch pathway modulators and chemotherapeutic agents, cells were seeded in 96 well-plates and cultured for 48 hours in presence of increasing concentrations of each compound. Then, the colorimetric assay with 3-[4,5-dimethylthiazol-2-yl]-2,5-diphenyltetrazolium bromide (MTT, Sigma-Aldrich) was performed, as previously described [34].

### Cell proliferation and viability assays

Cell proliferation was evaluated by flow cytometry after carboxy-fluorescein-succinimidyl ester (CFSE) staining (Life Technologies), as previously described [34]. Briefly, cells were washed twice with PBS and resuspended in 0.1% PBS-BSA, stained with CFSE (5 mM) for 10 minutes in the dark at 37° C and incubated 5 minutes on ice. Stained cells were used in different experiments. Relative cell proliferation was expressed as the percentage of CFSE median fluorescence (according to flow cytometric analysis) of treated cells compared to that of cells treated with the specific vehicle. Cell viability was assessed by TOPRO-3 staining, as previously described [34].

### Apoptosis

Apoptotic rate of B-cells was assessed using FITC-Annexin V/Propidium Iodide (PI) staining, as previously described. Briefly, B-ALL cells were washed twice with PBS and then stained with APC-conjugated anti-CD19 for 15 minutes in the dark at room temperature. Cells were re-suspended in binding buffer (MiltenyiBiotec), and FITC-conjugated Annexin V (MiltenyiBiotec) was added at 1 μg/mL final concentration. The mixture was incubated at room temperature for 15 minutes in the dark. Membrane integrity was assessed by PI staining, immediately before flow cytometric analysis, by using a FACS Canto II (BD Biosciences).

### Xenograft mouse model

NOD/Shi-scid/IL-2Rγnull (NOG) mice were purchased from Taconic (Germantown, NY) and kept in pathogen-free conditions in the animal facility of the Interdepartmental Centre of Experimental Research Service (CIRSAL) of the University of Verona, as approved by the Italian Minister of Health. VR-ALL cells (5 × 10^6^) were injected via tail vein into 8 to 12 weeks old mice previously irradiated with 1.2 Gy from a ^137^Cs source. Eight weeks following the initial injection of VR-ALL cells, animals were sacrificed and leukemic burden was quantified in organs as number of hCD45+ cells.

### Genomic sequencing and analysis

Genomic sequencing and analysis are detailed in Supplementary Methods.

## SUPPLEMENTARY MATERIALS


