# Supplementary material for: Characterization of a new B-ALL cell line with constitutional defect of the Notch signaling pathway
Source: Oncotarget. 2018 Apr 6;9(26):18341–50. doi: 10.18632/oncotarget.24836 (PMC5915076; doi:10.18632/oncotarget.24836)
Supplement: Supplementary file 1 [file oncotarget-09-18341-s001.pdf]

Characterization of a new B-ALL cell line with constitutional defect of the Notch signaling pathway

SUPPLEMENTARY MATERIALS

Chemicals and antibodies

The antibodies used for FACS analysis were: mouse IgG2b-FITC, goat IgG-PE, anti-Jagged1-FITC, anti-Dll3- PE (all from R&D System, Minneapolis, MN), mouse IgG2a-PE, mouse IgG1κ-PE, mouse IgG1-Alexa Fluor 488, anti-Notch1-PE, anti-Notch2-PE, anti-Notch3-PE, anti-Notch4-PE, anti-Dll1-PE, anti-Dll4, (all from Biolegend, San Diego, CA). For leukemia B-cells identification we used anti-CD19-PerCp/-APC and anti-CD45-PerCp/-APC/-V450/V500 (all from Miltenyi Biotec, Germany). The antibodies employed for western blot analysis anti-Notch2, anti-Notch4 were from Santa Cruz (Biotechnology, Dallas, TX), anti-GAPDH and HRP conjugated secondary antibodies against mouse, rabbit or goat were from Sigma Aldrich. All the other antibodies used for Western blot were from Cell Signaling. Neutralizing antibodies, all used at a final concentration of 5 µg/ml, were: anti-Notch1,anti-Notch3, anti-Notch2 (R&D Systems); anti-Notch4 (Santa Cruz Biotechnology). GSI-IX (DAPT) was purchased from Stemgent (Cambridge, MA) GSI-XII were from Merck Millipore (Darmstadt, Germany). Cytarabine (Ara-C), Dexamethasone (Dexa), Doxorubicin (Doxo), Bortezomid and MG132 were provided by Pharmacy Unit of the University Hospital of Verona.

Genomic analysis

Cell samples were collected after written informed consent, as approved by the Ethics Committee of Azienda Ospedaliera Universitaria Integrata Verona (N. Prog. 1828, May 12, 2010 - 'Institution of cell and tissue collection for biomedical research in Onco-Hematology'). Mononuclear cells were purified by Ficoll-Paque centrifugation (Lymphoprep, Fresenius Kabi Norge AS for Axis-Shield Poc AS, Oslo, Norway), washed in phosphate-buffered saline solution (PBS) and resuspended at 10×10^6/mL concentration in PBS. DNA was obtained from 10^7 cells by GentraPuregene cell kit (Qiagen, Hilden, Germany). Genomic DNA was quantified using the Quant-iTPicoGreen dsDNA Assay Kit (Life Technologies, Carlsbad, CA, USA) on a NanoDrop 3300 fluorospectrometer (Thermo Scientific, Wilmington, DE, USA). Then, 1.3 µg of DNA was sheared to 100–450 bp on a Covaris S220 instrument (Covaris, Woburn, Massachusetts, USA). Fragmentation was verified on the Agilent 2100 Bioanalyzer using a DNA 1000 assay (Agilent, Santa Clara, CA, USA). Sheared DNA was subjected to Illumina paired-end DNA library preparation using the TruSeq DNA Sample preparation kit (Illumina, San Diego, CA, USA). The quality of the library was evaluated with an Agilent High Sensitivity DNA assay (Agilent, Santa Clara, CA, USA) and then quantified using the Quant-iTPicoGreen dsDNA Assay Kit (Life Technologies, Carlsbad, CA, USA) on a NanoDrop 3300 fluorospectrometer (Thermo Scientific, Wilmington, DE, USA). Exome capture was performed starting from 500 ng of library as input material with TruSeq Exome Enrichment Kit (Illumina, San Diego, CA, USA) following manufacturer instruction. The quality of the whole exome library was checked with an Agilent High Sensitivity DNA assay and quantified by qPCR on a Stratagene MX3000P (Agilent, Santa Clara, CA, USA) using Kapa Library Quant kit (Kapa Biosystems, Woburn, MA). Whole exome library was sequenced with an Illumina HiSeq 1000 sequencer (Illumina Inc., San Diego, CA, USA) and 100-bp paired-end sequences were generated. Putative pathogenic variants identified in Notch genes were confirmed by Sanger sequencing. Raw reads were processed by Knome Inc. using hg19 human genome assembly as a reference. Called annotated variants were filtered using knome VARIANTS software. "Intron", "synonymous", "non-genic" and "untranslated" options were deselected and variants passing the quality filter were selected ("Passes quality filter" option). Only variants with a phred scaled call confidence >50 were considered and a threshold on allele frequency <0.05 was set. Potentially damaging variants were selected by setting a minimum effect score threshold of 0.469 and evolutionary conserved variants were selected by setting a conservation score ≥0.8. Next, 2 systems biology–ranking algorithms, Endeavor (http://homes.esat.kuleuven.be/≈bioiuser/endeavour/tool/endeavourweb/php), and ToppGene (http://toppgene.cchmc.org/prioritization.jsp), were used to rank the putative disease-causative genes. The softwares were trained with a custom panel of 44 genes known to be involved or related to ALL. Default prioritization parameters were used for each algorithm.
